# Supplementary material for: The Gut–Brain–Microbiome Axis in Bumble Bees
Source: Insects. 2020 Aug 10;11(8):517. doi: 10.3390/insects11080517 (PMC7469151; doi:10.3390/insects11080517)

FMPER Stats_Revised

Laura Leger

July 21, 2020

```
#setwd("~/Bumble bee cognition")

#import dataset
#install.packages("readxl")
library(readxl)
#install.packages("tidyverse")
library(tidyverse)
```

```
## -- Attaching packages ------------------------------------------------------------------------

## v ggplot2 3.3.2     v purrr   0.3.4
## v tibble  3.0.3     v dplyr   1.0.0
## v tidyr   1.1.0     v stringr 1.4.0
## v readr   1.3.1     v forcats 0.5.0

## -- Conflicts ---------------------------------------------------------------------------------
## x dplyr::filter() masks stats::filter()
## x dplyr::lag()    masks stats::lag()
```

```
#install.packages("devtools")

#read sheets in excel file
D0 <- read_excel("FMPER_data2_Fall2018.xlsx", #sheet = "Sheet1",
                na = c("", "NA", "N/A"))
#str(D0)
read_excel_allsheets <- function(filename) {
  sheets <- readxl::excel_sheets(filename)
  x <-    lapply(sheets, function(X) readxl::read_excel(filename, sheet = X)) ##insert na.strings parame
  names(x) <- sheets
  x
}

#bind and label sheets to use
#list.files()
D<-read_excel_allsheets("FMPER_data2_Fall2018.xlsx")
```

```
## New names:
## * `` -> ...7

## New names:
## * `` -> ...7

## New names:
## * `` -> ...8
## * `` -> ...9
```

```
## New names:
## * `` -> ...1
## * `` -> ...2
## * `` -> ...3
## * `` -> ...4
## * `` -> ...9
## * ...
```

```
#str(D)
D_choice<- rbind(D$choice_score)
#str(D_choice)

D_part<- rbind(D$participation_score)
#str(D_part)
```

```
#participation analyses for all experimental rounds
#install.packages("lme4")
library(lme4)
```

```
## Loading required package: Matrix

##
## Attaching package: 'Matrix'

## The following objects are masked from 'package:tidyr':
##
##     expand, pack, unpack
```

```
#install.packages("blme")
library(blme)
#install.packages("broom")
library(broom)


#goodness of fit tests for participation model
#asses different variations of model to find the best fit
mp1<-bglmer(part_response ~ treatment + expmt_round + (1|colony) + (1|age), data = D_part, family = bino

mp2<-bglmer(part_response ~ treatment + expmt_round + (1|colony), data = D_part, family = binomial(link

mp3<-bglmer(part_response ~ treatment + expmt_round + (1|age), data = D_part, family = binomial(link =

mp4<-glm(part_response ~ treatment + expmt_round, data = D_part, family = binomial(link = "logit"))


AIC(mp1)
```

```
## [1] 169.2693
```

```
#169.2692
#e^[(163.2612-169.2692)/2] = 0.0496; we reject this model
AIC(mp2)
```

```
## [1] 164.3984
```

```
#164.3984
#e^[(163.2612-164.3984)/2] = 0.5663; likely accept this model, though either this or mp4 would be accep
AIC(mp3)
```

```
## [1] 167.756
```

```
#167.756
#e^[(163.2612-167.756)/2] = 0.1057; we reject this model
AIC(mp4)
```

```
## [1] 163.2612
```

```
#163.2612; lowest AIC of all model choices

#settle on a model that excludes age, but includes colony as a random effect
part_fit<-bglmer(part_response ~ treatment + expmt_round + (1|colony), data = D_part, family = binomial
summary(part_fit)
```

```
## Cov prior  : colony ~ wishart(df = 3.5, scale = Inf, posterior.scale = cov, common.scale = TRUE)
## Prior dev  : 0.6256
##
## Generalized linear mixed model fit by maximum likelihood (Laplace
##   Approximation) [bglmerMod]
##  Family: binomial  ( logit )
## Formula: part_response ~ treatment + expmt_round + (1 | colony)
##    Data: D_part
##
##      AIC      BIC   logLik deviance df.resid
##    164.4    175.9    -78.2    156.4      127
##
## Scaled residuals:
##     Min      1Q  Median      3Q     Max
## -2.8078 -1.0851  0.4484  0.6482  0.9732
##
## Random effects:
##  Groups Name        Variance Std.Dev.
##  colony (Intercept) 0.659    0.8118
## Number of obs: 131, groups:  colony, 9
##
## Fixed effects:
##                             Estimate Std. Error z value Pr(>|z|)
## (Intercept)                   1.0521     1.1957   0.880    0.379
## treatmentMicrobe_Inoculated   0.3744     0.4111   0.911    0.362
## expmt_round                  -0.1098     0.7043  -0.156    0.876
##
## Correlation of Fixed Effects:
##             (Intr) trtM_I
## trtmntMcr_I -0.137
## expmt_round -0.943 -0.011
```

```
##choice analyses with likelihood ratio tests for random variables
#generalized linear model with mixed effects on choice response in assay

#goodness of fit tests for choice model
#asses different variations of model to find the best fit
mc1<-bglmer(choice_response ~ treatment + expmt_round + (1|colony) + (1|age), data = D_choice, family =

mc2<-bglmer(choice_response ~ treatment + expmt_round + (1|colony), data = D_choice, family = binomial(l

mc3<-bglmer(choice_response ~ treatment + expmt_round + (1|age), data = D_choice, family = binomial(lin
```

```r
mc4<-glm(choice_response ~ treatment + expmt_round, data = D_choice, family = binomial(link = "logit"))

AIC(mc1)
```

```
## [1] 134.2952
```

```r
#134.2953
#e^[(125.9927 - 134.2953)/2] = 0.0157, we reject this model
AIC(mc2)
```

```
## [1] 129.6095
```

```r
#129.6094
#e^[(125.9927 - 129.6094)/2] = 0.1639; potentially reject this model
AIC(mc3)
```

```
## [1] 130.4562
```

```r
#130.4562
#e^[(125.9927 - 130.4562)/2] = 0.1073; we reject this model
AIC(mc4)
```

```
## [1] 125.9927
```

```r
#125.9927
#lowest AIC of model choices

#settle on a model that excludes age, but includes colony as a random effect
choice_fit<-bglmer(choice_response ~ treatment + expmt_round + (1|colony), data = D_choice, family = bi
summary(choice_fit)
```

```
## Cov prior  : colony ~ wishart(df = 3.5, scale = Inf, posterior.scale = cov, common.scale = TRUE)
## Prior dev  : 2.8159
##
## Generalized linear mixed model fit by maximum likelihood (Laplace
##   Approximation) [bglmerMod]
##  Family: binomial  ( logit )
## Formula: choice_response ~ treatment + expmt_round + (1 | colony)
##    Data: D_choice
##
##      AIC      BIC   logLik deviance df.resid
##    129.6    139.7    -60.8    121.6       89
##
## Scaled residuals:
##     Min      1Q  Median      3Q     Max
## -1.5959 -1.1481  0.6395  0.7172  0.9100
##
## Random effects:
##  Groups Name        Variance Std.Dev.
##  colony (Intercept) 0.153    0.3912
## Number of obs: 93, groups:  colony, 9
##
## Fixed effects:
##                             Estimate Std. Error z value Pr(>|z|)
## (Intercept)                 -0.16238    0.90537  -0.179    0.858
## treatmentMicrobe_Inoculated -0.00677    0.45011  -0.015    0.988
## expmt_round                  0.48535    0.52551   0.924    0.356
```

```
##
## Correlation of Fixed Effects:
##              (Intr) trtM_I
## trtmntMcr_I -0.286
## expmt_round -0.924  0.033
```

```r
#demonstrate that FMPER assay is appropriate using raw choice data from experiment:

#participation data ignoring treatment including "neither color"
part<- matrix(c(93, 38), ncol=1)
colnames(part)<-c('Number of Bees')
rownames(part)<-c('Participated', 'Did Not Participate')
part.table1<-as.table(part)
part.table1
```

```
##                       Number of Bees
## Participated                      93
## Did Not Participate               38
```

```r
chisq.test(part.table1)
```

```
##
##  Chi-squared test for given probabilities
##
## data:  part.table1
## X-squared = 23.092, df = 1, p-value = 1.545e-06
```

```r
#chi-squared test for participation by treatment without random variables
part2<- matrix(c(45, 21, 48, 17), ncol=2)
colnames(part2)<-c('Microbe-Depleted', 'Microbe-Inoculated')
rownames(part2)<-c('Participated', 'Did Not Participate')
part.table2<-as.table(part2)
part.table2
```

```
##                       Microbe-Depleted Microbe-Inoculated
## Participated                        45                 48
## Did Not Participate                 21                 17
```

```r
chisq.test(part.table2)
```

```
##
##  Pearson's Chi-squared test with Yates' continuity correction
##
## data:  part.table2
## X-squared = 0.27224, df = 1, p-value = 0.6018
```

```r
#choice data ignoring treatment with "neither color" removed
choice.all<-matrix(c(60, 33), ncol=1)
colnames(choice.all)<-c('Number of Bees')
rownames(choice.all)<-c('Trained Color', "Novel COlor")
choice.all.table<-as.table(choice.all)
choice.all.table
```

```
##                 Number of Bees
## Trained Color               60
## Novel COlor                 33
```

```r
chisq.test(choice.all.table)
```

```
##
##  Chi-squared test for given probabilities
##
## data:  choice.all.table
## X-squared = 7.8387, df = 1, p-value = 0.005114
```

```r
#chi-squared test for choice by treatment without random variables
choice2<-matrix(c(29, 16, 31, 17), ncol=2)
colnames(choice2)<-c('Microbe-Depleted', 'Microbe-Inoculated')
rownames(choice2)<-c('Trained Color', "Novel COlor")
choice.table2<-as.table(choice2)
choice.table2
```

```
##               Microbe-Depleted Microbe-Inoculated
## Trained Color               29                 31
## Novel COlor                 16                 17
```

```r
chisq.test(choice.table2)
```

```
##
##  Pearson's Chi-squared test with Yates' continuity correction
##
## data:  choice.table2
## X-squared = 1.9761e-31, df = 1, p-value = 1
```

```r
#calculating binomial confidence intervals from raw data
library(binom)
library(Hmisc)
```

```
## Loading required package: lattice

## Loading required package: survival

## Loading required package: Formula

##
## Attaching package: 'Hmisc'

## The following objects are masked from 'package:dplyr':
##
##     src, summarize

## The following objects are masked from 'package:base':
##
##     format.pval, units
```

```r
#participation data
binom.pmd<-binom.logit(45, 66, conf.level = 0.95)
binom.pmd
```

```
##   method  x  n      mean     lower     upper
## 1  logit 45 66 0.6818182 0.5607412 0.7824684
```

```r
#method  x   n    mean      lower     upper
#logit   45 66  0.6818182   0.5607412   0.7824684

binconf(x = 45, n = 66)
```

```
##   PointEst     Lower     Upper
##  0.6818182 0.5621255 0.7815099
```

```r
#  PointEst     Lower     Upper
# 0.6818182 0.5621255 0.7815099

binom.pmi<-binom.logit(48, 65, conf.level = 0.95)
binom.pmi
```

```
##   method  x  n      mean     lower     upper
## 1  logit 48 65 0.7384615 0.6188845 0.8307791
```

```r
#method n   x   mean      lower     upper
#logit  48  65  0.7384615   0.6188845   0.8307791

binconf(x = 48, n = 65)
```

```
##   PointEst    Lower     Upper
##  0.7384615 0.620492 0.8298181
```

```r
#  PointEst    Lower     Upper
# 0.7384615 0.620492 0.8298181

#choice data
binom.cmd<-binom.logit(29, 45, conf.level = 0.95)
binom.cmd
```

```
##   method  x  n      mean     lower     upper
## 1  logit 29 45 0.6444444 0.4960836 0.7694273
```

```r
#method n   x   mean      lower     upper
#logit  29  45  0.6444444   0.4960836   0.7694273


binconf(x = 29, n = 45)
```

```
##   PointEst     Lower     Upper
##  0.6444444 0.4983581 0.7678092
```

```r
#  PointEst     Lower     Upper
# 0.6444444 0.4983581 0.7678092

binom.cmi<-binom.logit(31, 48, conf.level = 0.95)
binom.cmi
```

```
##   method  x  n      mean     lower     upper
## 1  logit 31 48 0.6458333 0.5023155 0.7671496
```

```r
#method n   x   mean      lower     upper
#logit  31  48  0.6458333   0.5023155   0.7671496


binconf(x = 31, n = 48)
```

```
##   PointEst     Lower     Upper
##  0.6458333 0.5043906 0.7656636
```

```r
#  PointEst     Lower     Upper
# 0.6458333 0.5043906 0.7656636
```

```
library(ggplot2)
##install.packages("scales")
library(scales)
```

```
##
## Attaching package: 'scales'

## The following object is masked from 'package:purrr':
##
##     discard

## The following object is masked from 'package:readr':
##
##     col_factor
```

```
##install.packages("Hmisc")
library(Hmisc)

#graphing results with barplot for participation using proportions from raw data

#plot with ggplot, calculate CI inside of plot code
part<-ggplot(D_part, aes(treatment, part_response)) +
  stat_summary(fun=mean, geom="bar", fill="grey70") +
  stat_summary(fun.data=mean_cl_boot, geom="errorbar", width=0.2) + ##this line calculates CI using boo
  scale_y_continuous(labels=percent_format(), limits=c(0,1)) +
  theme_bw()
print(part + labs(title= "Bumble Bee Participation in Assay (Combined Rounds)",
                      y="Proportion of Participation in Assay", x = "Treatment"))
```

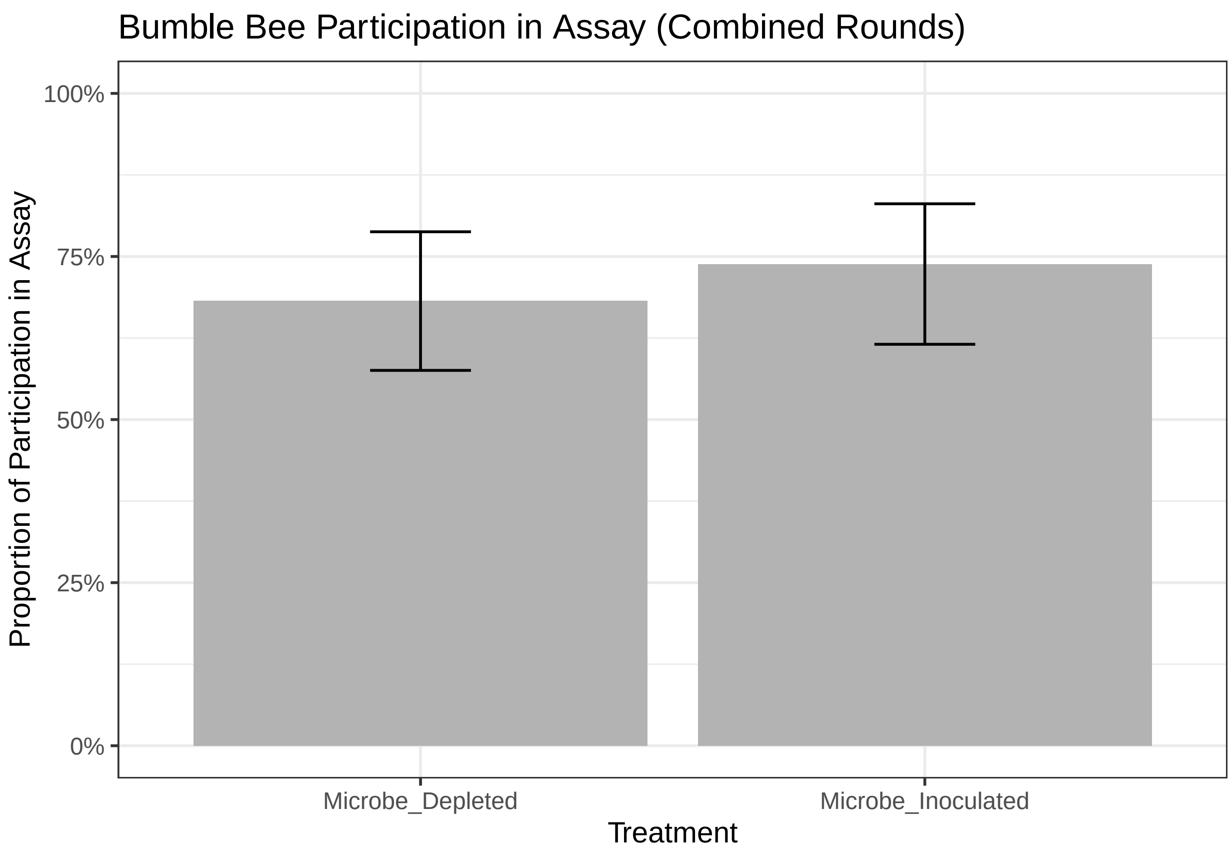


```
#graphing results with barplot for choice using proportions from raw data

#using ggplot to graph results with CI calc included in code
choice.ggplot<-ggplot(D_choice, aes(treatment, choice_response)) +
  stat_summary(fun=mean, geom="bar", fill="grey70") +
  stat_summary(fun.data=mean_cl_boot, geom="errorbar", width=0.2) + ##this line calculates CI with boots
  scale_y_continuous(labels=percent_format(), limits=c(0,1)) +
  theme_bw()
print(choice.ggplot + labs(title= "Bumble Bee Choice Response in Assay (Combined Rounds)",
                           y="Proportion of Response to Trained Color", x = "Treatment"))
```

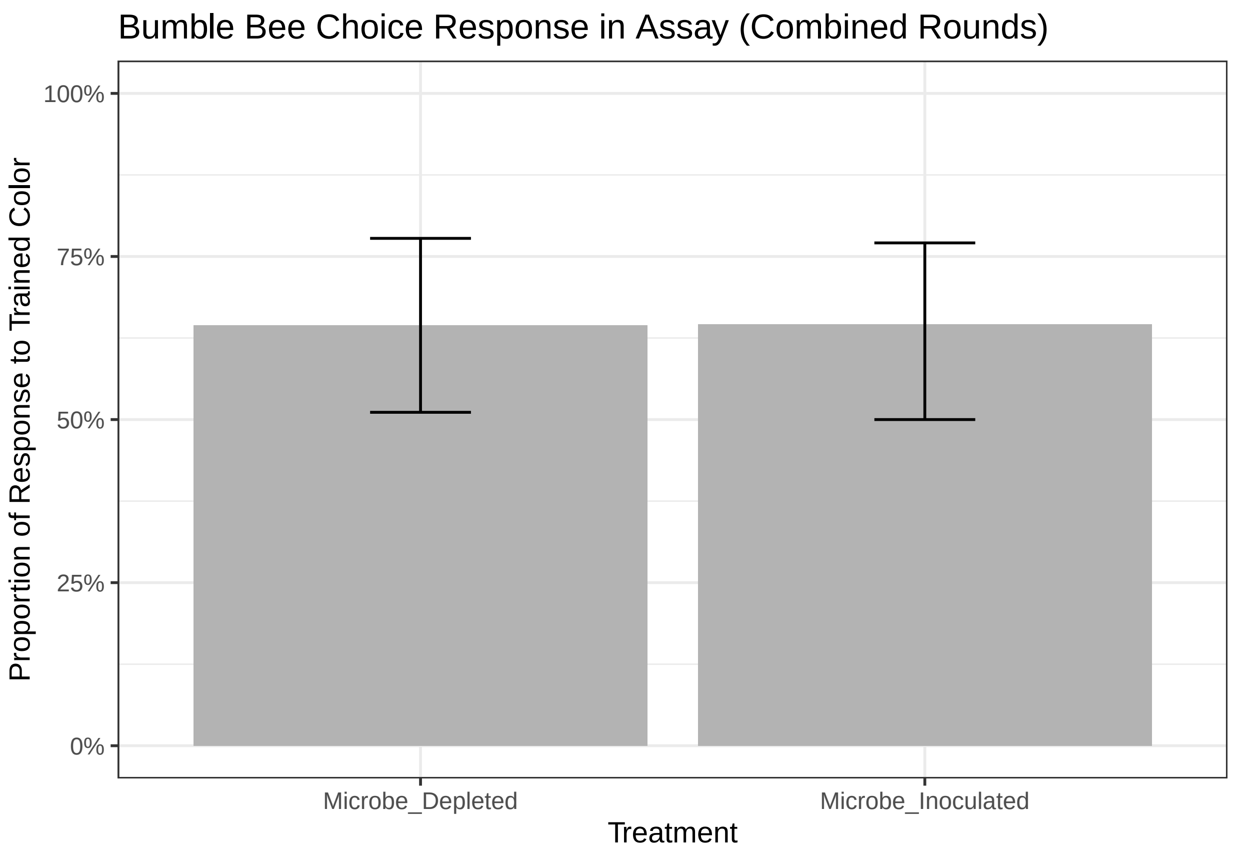


```
##Survival Stats Using Cox Proportional Hazards
library(coxme)
```

```
## Loading required package: bdsmatrix

##
## Attaching package: 'bdsmatrix'

## The following object is masked from 'package:base':
##
##     backsolve
```

```
library(survival)
#install.packages("survminer")
library(survminer)
```

```
## Loading required package: ggpubr

## Registered S3 methods overwritten by 'car':
##   method                          from
##   influence.merMod                lme4
##   cooks.distance.influence.merMod lme4
##   dfbeta.influence.merMod         lme4
##   dfbetas.influence.merMod        lme4
```

```
library(readxl)
```

```r
#read sheets in excel file
D0 <- read_excel("survival_data_indiv_Fall2018.xlsx", #sheet = "Sheet1",
                 na = c("", "NA", "N/A"))
#str(D0)
read_excel_allsheets <- function(filename) {
  sheets <- readxl::excel_sheets(filename)
  x <-    lapply(sheets, function(X) readxl::read_excel(filename, sheet = X)) ##insert na.strings parame
  names(x) <- sheets
  x
}

#bind and label sheets to use
#list.files()
D1<-read_excel_allsheets("survival_data_indiv_Fall2018.xlsx")
#str(D1)
D1_all<- rbind(D1$round1)
#str(D1_all)

D2<-read_excel_allsheets("survival_data_indiv_Fall2018.xlsx")
#str(D2)
D2_all<- rbind(D2$round2)
#str(D2_all)


#round 1 model and graph
data1<-coxph(formula = Surv(day, mortality) ~ treatment + colony, data = D1_all)
```

```
## Warning in fitter(X, Y, istrat, offset, init, control, weights = weights, :
## Loglik converged before variable 2,3,5 ; coefficient may be infinite.
```

```r
#To check if the data violates cox proportionality (you want p>0.05)
prop<-cox.zph(data1)
prop
```

```
##               chisq df    p
## treatment 0.000276  1 0.99
## colony    2.144136  4 0.71
## GLOBAL    2.170425  5 0.83
```

```r
#             chisq df    p
#treatment 0.000276  1 0.99
#colony    2.144136  4 0.71
#GLOBAL    2.170425  5 0.83

#mixed effects model for round 1
data1me<-coxme(formula = Surv(day, mortality) ~ treatment + (1|colony), data = D1_all)
data1me
```

```
## Cox mixed-effects model fit by maximum likelihood
##   Data: D1_all
##   events, n = 3, 58
##   Iterations= 2 10
##                     NULL Integrated    Fitted
## Log-likelihood -12.12885  -12.04388 -12.04308
##
```

```
##                     Chisq df       p   AIC   BIC
## Integrated loglik  0.17  2 0.91855 -3.83 -2.03
##  Penalized loglik  0.17  1 0.67910 -1.83 -0.93
##
## Model:  Surv(day, mortality) ~ treatment + (1 | colony)
## Fixed coefficients
##                    coef exp(coef) se(coef)    z    p
## treatmentS -0.4935442  0.610459   1.2248 -0.4 0.69
##
## Random effects
##  Group  Variable  Std Dev      Variance
##  colony Intercept 0.0199991940 0.0003999678
```

```
#round 2 model and graph
data2<-coxph(formula=Surv(day, mortality) ~ treatment + colony, data = D2_all)
data2
```

```
## Call:
## coxph(formula = Surv(day, mortality) ~ treatment + colony, data = D2_all)
##
##                coef exp(coef) se(coef)      z       p
## treatmentS -0.7446    0.4749   0.3403 -2.188 0.02863
## colonyL2    1.6274    5.0908   1.0802  1.507 0.13191
## colonyL3    0.6448    1.9056   1.2248  0.526 0.59856
## colonyL4    2.2352    9.3480   1.0694  2.090 0.03660
## colonyL6    2.9624   19.3434   1.0232  2.895 0.00379
##
## Likelihood ratio test=36.61  on 5 df, p=7.162e-07
## n= 163, number of events= 39
```

```
#To check if the data violates cox proportionality (you want p>0.05)
prop2<-(cox.zph(data2))
prop2
```

```
##            chisq df     p
## treatment  0.122  1 0.727
## colony    11.293  4 0.023
## GLOBAL    11.389  5 0.044
```

```
#            chisq df     p
#treatment  0.122  1 0.727
#colony    11.293  4 0.023
#GLOBAL    11.389  5 0.044
##seems that colony and global data violate the assumptions of CPH - what to do?
#cannot use coxph methods, repeat with Kaplan-Meier assessment

#mixed effects model for round 2
data2me<-coxme(formula = Surv(day, mortality) ~ treatment + (1|colony), data = D2_all)
data2me
```

```
## Cox mixed-effects model fit by maximum likelihood
##   Data: D2_all
##   events, n = 39, 163
##   Iterations= 5 24
##                       NULL Integrated    Fitted
## Log-likelihood -193.1797  -180.5524 -175.2512
##
```

```
##                    Chisq   df          p   AIC   BIC
## Integrated loglik 25.25 2.00 3.2813e-06 21.25 17.93
##  Penalized loglik 35.86 4.31 4.5512e-07 27.23 20.06
##
## Model:  Surv(day, mortality) ~ treatment + (1 | colony)
## Fixed coefficients
##                   coef exp(coef)  se(coef)     z     p
## treatmentS -0.7436048 0.4753971 0.3401805 -2.19 0.029
##
## Random effects
##  Group  Variable  Std Dev  Variance
##  colony Intercept 1.074262 1.154038
```

```
#install.packages("rlang")
#survival stats with kaplan-meier because data violate assumptions of CPH
library(readxl)
library(survival)
#install.packages("ranger")
library(ranger)
library(ggplot2)
library(dplyr)
#install.packages("ggfortify")
library(ggfortify)
#install.packages("survminer")
library(survminer)


#read sheets in excel file
D0 <- read_excel("survival_data_indiv_Fall2018.xlsx", #sheet = "Sheet1",
                na = c("", "NA", "N/A"))
str(D0)
```

```
## tibble [163 x 7] (S3: tbl_df/tbl/data.frame)
##  $ bee        : num [1:163] 1 2 3 4 5 6 7 8 9 10 ...
##  $ pot        : num [1:163] 1 1 1 1 1 1 1 2 2 2 ...
##  $ colony     : chr [1:163] "L3" "L3" "L3" "L3" ...
##  $ expmt_round: num [1:163] 2 2 2 2 2 2 2 2 2 2 ...
##  $ treatment  : chr [1:163] "S" "S" "S" "S" ...
##  $ day        : num [1:163] 8 8 8 8 8 8 8 8 8 8 ...
##  $ mortality  : num [1:163] 0 0 0 0 0 0 0 0 0 0 ...
```

```
read_excel_allsheets <- function(filename) {
  sheets <- readxl::excel_sheets(filename)
  x <-    lapply(sheets, function(X) readxl::read_excel(filename, sheet = X)) ##insert na.strings parame
  names(x) <- sheets
  x
}

#bind and label sheets to use
#list.files()
D1<-read_excel_allsheets("survival_data_indiv_Fall2018.xlsx")
str(D1)
```

```
## List of 2
##  $ round2: tibble [163 x 7] (S3: tbl_df/tbl/data.frame)
##   ..$ bee        : num [1:163] 1 2 3 4 5 6 7 8 9 10 ...
```

```
##   ..$ pot         : num [1:163] 1 1 1 1 1 1 1 2 2 2 ...
##   ..$ colony      : chr [1:163] "L3" "L3" "L3" "L3" ...
##   ..$ expmt_round: num [1:163] 2 2 2 2 2 2 2 2 2 2 ...
##   ..$ treatment  : chr [1:163] "S" "S" "S" "S" ...
##   ..$ day         : num [1:163] 8 8 8 8 8 8 8 8 8 8 ...
##   ..$ mortality  : num [1:163] 0 0 0 0 0 0 0 0 0 0 ...
##  $ round1: tibble [58 x 7] (S3: tbl_df/tbl/data.frame)
##   ..$ bee         : chr [1:58] "1A" "2A" "3A" "12A" ...
##   ..$ pot         : num [1:58] 1 1 1 3 3 3 3 3 3 3 ...
##   ..$ colony      : chr [1:58] "E18" "E18" "E18" "E17" ...
##   ..$ expmt_round: num [1:58] 1 1 1 1 1 1 1 1 1 1 ...
##   ..$ treatment  : chr [1:58] "M" "M" "M" "M" ...
##   ..$ day         : num [1:58] 8 8 8 4 8 8 8 8 8 8 ...
##   ..$ mortality  : num [1:58] 0 0 0 1 0 0 0 0 0 0 ...
```

```
D1_all<- rbind(D1$round1)
str(D1_all)
```

```
## tibble [58 x 7] (S3: tbl_df/tbl/data.frame)
##  $ bee         : chr [1:58] "1A" "2A" "3A" "12A" ...
##  $ pot         : num [1:58] 1 1 1 3 3 3 3 3 3 3 ...
##  $ colony      : chr [1:58] "E18" "E18" "E18" "E17" ...
##  $ expmt_round: num [1:58] 1 1 1 1 1 1 1 1 1 1 ...
##  $ treatment  : chr [1:58] "M" "M" "M" "M" ...
##  $ day         : num [1:58] 8 8 8 4 8 8 8 8 8 8 ...
##  $ mortality  : num [1:58] 0 0 0 1 0 0 0 0 0 0 ...
```

```
D2<-read_excel_allsheets("survival_data_indiv_Fall2018.xlsx")
str(D2)
```

```
## List of 2
##  $ round2: tibble [163 x 7] (S3: tbl_df/tbl/data.frame)
##   ..$ bee         : num [1:163] 1 2 3 4 5 6 7 8 9 10 ...
##   ..$ pot         : num [1:163] 1 1 1 1 1 1 1 2 2 2 ...
##   ..$ colony      : chr [1:163] "L3" "L3" "L3" "L3" ...
##   ..$ expmt_round: num [1:163] 2 2 2 2 2 2 2 2 2 2 ...
##   ..$ treatment  : chr [1:163] "S" "S" "S" "S" ...
##   ..$ day         : num [1:163] 8 8 8 8 8 8 8 8 8 8 ...
##   ..$ mortality  : num [1:163] 0 0 0 0 0 0 0 0 0 0 ...
##  $ round1: tibble [58 x 7] (S3: tbl_df/tbl/data.frame)
##   ..$ bee         : chr [1:58] "1A" "2A" "3A" "12A" ...
##   ..$ pot         : num [1:58] 1 1 1 3 3 3 3 3 3 3 ...
##   ..$ colony      : chr [1:58] "E18" "E18" "E18" "E17" ...
##   ..$ expmt_round: num [1:58] 1 1 1 1 1 1 1 1 1 1 ...
##   ..$ treatment  : chr [1:58] "M" "M" "M" "M" ...
##   ..$ day         : num [1:58] 8 8 8 4 8 8 8 8 8 8 ...
##   ..$ mortality  : num [1:58] 0 0 0 1 0 0 0 0 0 0 ...
```

```
D2_all<- rbind(D2$round2)
str(D2_all)
```

```
## tibble [163 x 7] (S3: tbl_df/tbl/data.frame)
##  $ bee         : num [1:163] 1 2 3 4 5 6 7 8 9 10 ...
##  $ pot         : num [1:163] 1 1 1 1 1 1 1 2 2 2 ...
##  $ colony      : chr [1:163] "L3" "L3" "L3" "L3" ...
##  $ expmt_round: num [1:163] 2 2 2 2 2 2 2 2 2 2 ...
```

```
##  $ treatment  : chr [1:163] "S" "S" "S" "S" ...
##  $ day        : num [1:163] 8 8 8 8 8 8 8 8 8 8 ...
##  $ mortality  : num [1:163] 0 0 0 0 0 0 0 0 0 0 ...
```

```
#expermental round 1
r1_fit <- surv_fit(Surv(day, mortality) ~ treatment, type = "kaplan-meier", conf.type = "log", data = D
summary(r1_fit)
```

```
## Call: survfit(formula = Surv(day, mortality) ~ treatment, data = structure(list(
##     bee = c("1A", "2A", "3A", "12A", "13A", "14A", "15A", "16A",
##     "17A", "18A", "19A", "20A", "21A", "22A", "23A", "24A", "25A",
##     "26A", "27A", "75A", "76A", "77A", "78A", "79A", "80A", "81A",
##     "82A", "109A", "110A", "111A", "112A", "113A", "36A", "37A",
##     "38A", "39A", "40A", "41A", "42A", "43A", "59A", "60A", "61A",
##     "62A", "63A", "64A", "69A", "70A", "71A", "72A", "73A", "74A",
##     "97A", "98A", "99A", "100A", "101A", "102A"), pot = c(1,
##     1, 1, 3, 3, 3, 3, 3, 3, 3, 3, 4, 4, 4, 4, 4, 4, 4, 4, 12,
##     12, 12, 12, 12, 12, 12, 12, 17, 17, 17, 17, 17, 6, 6, 6,
##     6, 6, 6, 6, 6, 9, 9, 9, 9, 9, 9, 11, 11, 11, 11, 11, 11,
##     15, 15, 15, 15, 15, 15), colony = c("E18", "E18", "E18",
##     "E17", "E17", "E17", "E17", "E17", "E17", "E17", "E17", "E16",
##     "E16", "E16", "E16", "E16", "E16", "E16", "E16", "E13", "E13",
##     "E13", "E13", "E13", "E13", "E13", "E13", "E13", "E13", "E13",
##     "E13", "E13", "E16", "E16", "E16", "E16", "E16", "E16", "E16",
##     "E16", "E17", "E17", "E17", "E17", "E17", "E17", "E13", "E13",
##     "E13", "E13", "E13", "E13", "E15", "E15", "E15", "E15", "E15",
##     "E15"), expmt_round = c(1, 1, 1, 1, 1, 1, 1, 1, 1, 1, 1,
##     1, 1, 1, 1, 1, 1, 1, 1, 1, 1, 1, 1, 1, 1, 1, 1, 1, 1, 1,
##     1, 1, 1, 1, 1, 1, 1, 1, 1, 1, 1, 1, 1, 1, 1, 1, 1, 1, 1,
##     1, 1, 1, 1, 1, 1, 1, 1, 1), treatment = c("M", "M", "M",
##     "M", "M", "M", "M", "M", "M", "M", "M", "M", "M", "M", "M",
##     "M", "M", "M", "M", "M", "M", "M", "M", "M", "M", "M", "M",
##     "M", "M", "M", "M", "M", "S", "S", "S", "S", "S", "S", "S",
##     "S", "S", "S", "S", "S", "S", "S", "S", "S", "S", "S", "S",
##     "S", "S", "S", "S", "S", "S", "S"), day = c(8, 8, 8, 4, 8,
##     8, 8, 8, 8, 8, 8, 8, 8, 8, 8, 8, 8, 8, 1, 8, 8, 8, 8,
##     8, 8, 8, 8, 8, 8, 8, 8, 8, 8, 8, 8, 8, 8, 8, 8, 8, 8, 8,
##     8, 8, 8, 3, 8, 8, 8, 8, 8, 8, 8, 8, 8, 8, 8), mortality = c(0,
##     0, 0, 1, 0, 0, 0, 0, 0, 0, 0, 0, 0, 0, 0, 0, 0, 0, 0, 1,
##     0, 0, 0, 0, 0, 0, 0, 0, 0, 0, 0, 0, 0, 0, 0, 0, 0, 0, 0,
##     0, 0, 0, 0, 0, 0, 0, 1, 0, 0, 0, 0, 0, 0, 0, 0, 0, 0, 0)), row.names = c(NA,
## -58L), class = c("tbl_df", "tbl", "data.frame")), type = "kaplan-meier",
##     conf.type = "log")
##
##                 treatment=M
##  time n.risk n.event survival std.err lower 95% CI upper 95% CI
##     1     32       1    0.969  0.0308        0.910            1
##     4     31       1    0.938  0.0428        0.857            1
##
##                 treatment=S
##         time       n.risk      n.event     survival      std.err lower 95% CI
##       3.0000      26.0000       1.0000       0.9615       0.0377       0.8904
## upper 95% CI
##       1.0000
```

```
plot(r1_fit, main=expression(paste("Kaplan-Meier-estimate ", " with CI")), xlab="Time", ylab="Survival"
```

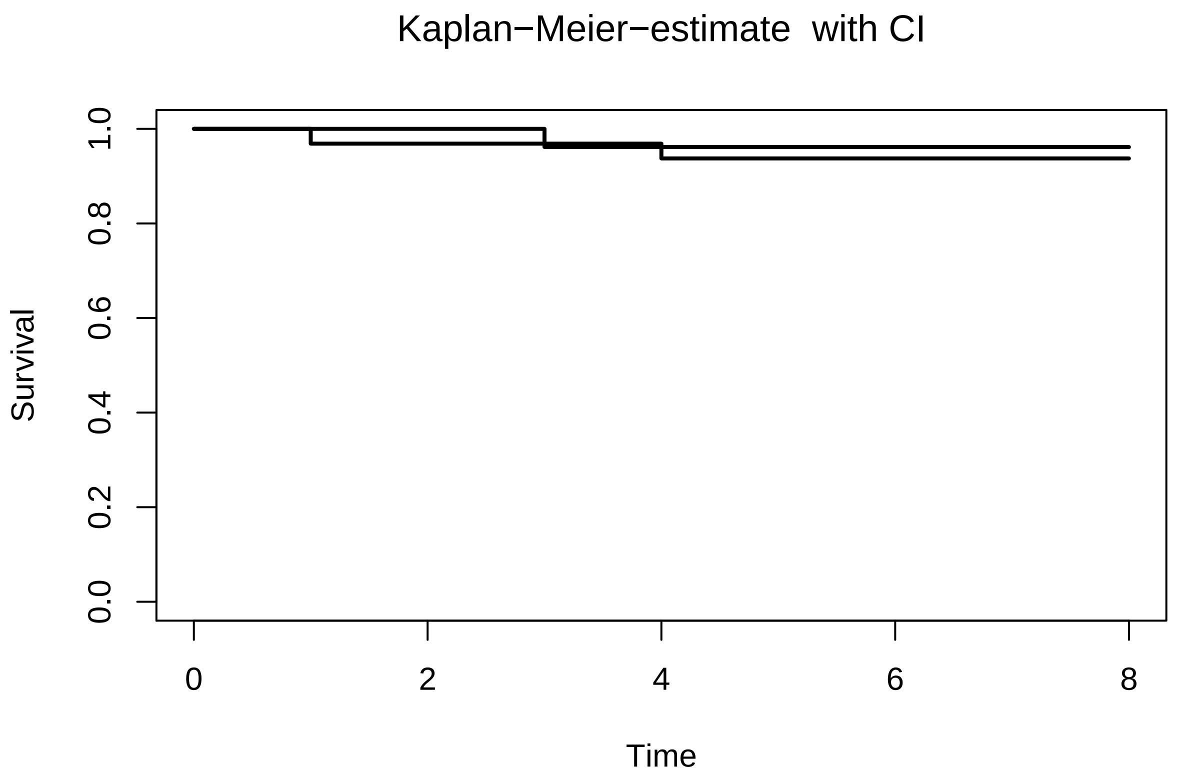


```
surv_diff1<-survdiff(Surv(day, mortality)~treatment + colony, data = D1_all)
surv_diff1
```

```
## Call:
## survdiff(formula = Surv(day, mortality) ~ treatment + colony,
##     data = D1_all)
##
##                            N Observed Expected (O-E)^2/E (O-E)^2/V
## treatment=M, colony=E13 13        1    0.649     0.190     0.242
## treatment=M, colony=E16  8        0    0.421     0.421     0.490
## treatment=M, colony=E17  8        1    0.421     0.796     0.926
## treatment=M, colony=E18  3        0    0.158     0.158     0.167
## treatment=S, colony=E13  6        1    0.298     1.654     1.837
## treatment=S, colony=E15  6        0    0.316     0.316     0.353
## treatment=S, colony=E16  8        0    0.421     0.421     0.490
## treatment=S, colony=E17  6        0    0.316     0.316     0.353
##
##  Chisq= 4.3  on 7 degrees of freedom, p= 0.7
```

```
#experimental round 2
r2<-survfit(Surv(day, mortality) ~  treatment,  type="kaplan-meier", conf.type="log", data=D2_all)
summary(r2)
```

```
## Call: survfit(formula = Surv(day, mortality) ~ treatment, data = D2_all,
##     type = "kaplan-meier", conf.type = "log")
```

```
##
##                 treatment=M
##  time n.risk n.event survival std.err lower 95% CI upper 95% CI
##     1     83       2    0.976  0.0168        0.943        1.000
##     2     81       6    0.904  0.0324        0.842        0.969
##     3     75       4    0.855  0.0386        0.783        0.935
##     5     71       4    0.807  0.0433        0.727        0.897
##     6     67       5    0.747  0.0477        0.659        0.847
##     7     62       3    0.711  0.0498        0.620        0.815
##     8     53       2    0.684  0.0514        0.590        0.792
##
##                 treatment=S
##  time n.risk n.event survival std.err lower 95% CI upper 95% CI
##     2     80       3    0.963  0.0212        0.922        1.000
##     4     77       3    0.925  0.0294        0.869        0.985
##     5     74       2    0.900  0.0335        0.837        0.968
##     6     72       2    0.875  0.0370        0.805        0.951
##     7     66       1    0.862  0.0387        0.789        0.941
##     8     63       2    0.834  0.0420        0.756        0.921
```

```
plot(r2, main=expression(paste("Kaplan-Meier-estimate ", " with CI")), xlab="Time", ylab="Survival", lw
```

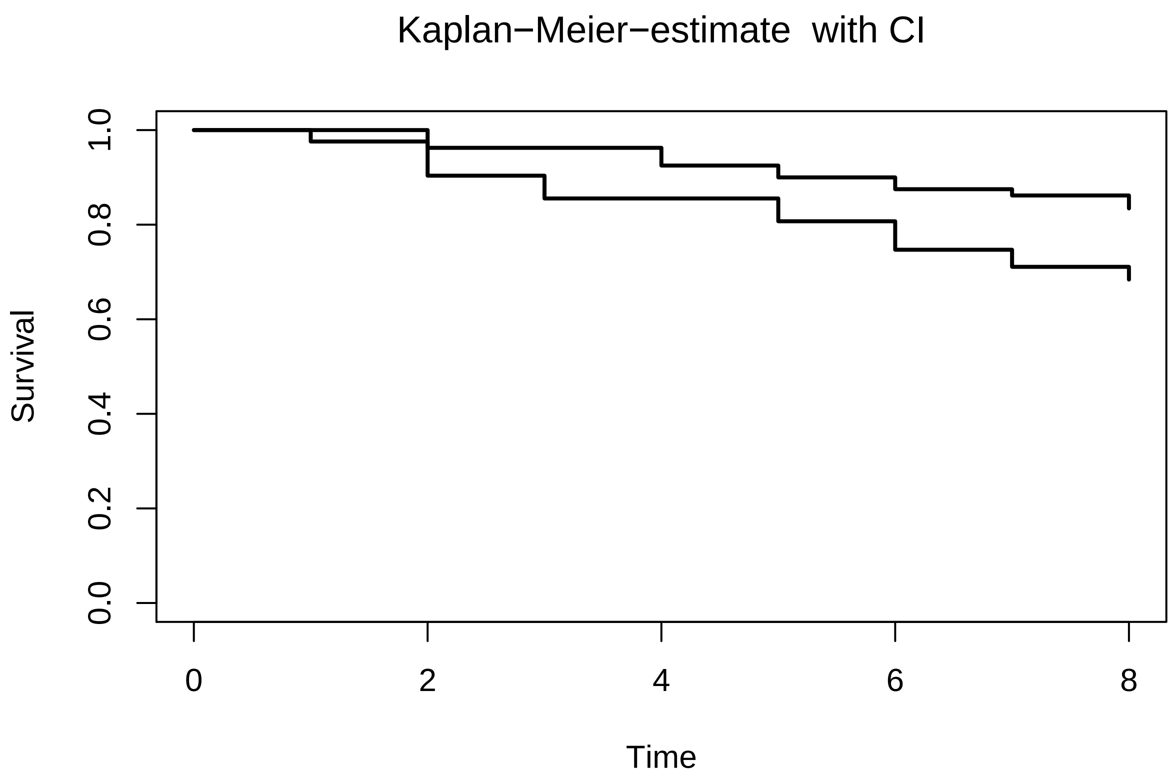


```
surv_diff2<-survdiff(Surv(day, mortality)~treatment + colony, data = D2_all)
surv_diff2
```

```
## Call:
## survdiff(formula = Surv(day, mortality) ~ treatment + colony,
```

```
##     data = D2_all)
##
##                          N Observed Expected (O-E)^2/E (O-E)^2/V
## treatment=M, colony=L1 13        1     3.54     1.822     2.074
## treatment=M, colony=L2 17        3     4.16     0.322     0.372
## treatment=M, colony=L3 14        1     3.81     2.074     2.379
## treatment=M, colony=L4 12        6     2.74     3.876     4.318
## treatment=M, colony=L6 27       15     4.79    21.769    25.946
## treatment=S, colony=L1 14        0     3.81     3.811     4.373
## treatment=S, colony=L2 18        3     4.73     0.635     0.748
## treatment=S, colony=L3 14        1     3.81     2.074     2.379
## treatment=S, colony=L4 11        1     2.99     1.328     1.489
## treatment=S, colony=L6 23        8     4.61     2.488     2.934
##
##  Chisq= 42.3  on 9 degrees of freedom, p= 3e-06
```

```r
#colony 6 over-represented in terms of sample size - maybe remove from both analyses?

r2<-survfit(Surv(day, mortality) ~  treatment,  type="kaplan-meier", conf.type="log", data=D2_all)

plot(r2, main=expression(paste("Kaplan-Meier-estimate ", " with CI")), xlab="Time", ylab="Survival", lwd
```

```r
#better graphs for survival data with Kaplan-Meier curves with confidence intervals
ggsurvplot(r2, conf.int=TRUE, title=expression(paste("Survival: Microbe-Inoculated vs. Microbe-Depleted
```

```
## Warning: Duplicated aesthetics after name standardisation: size
```

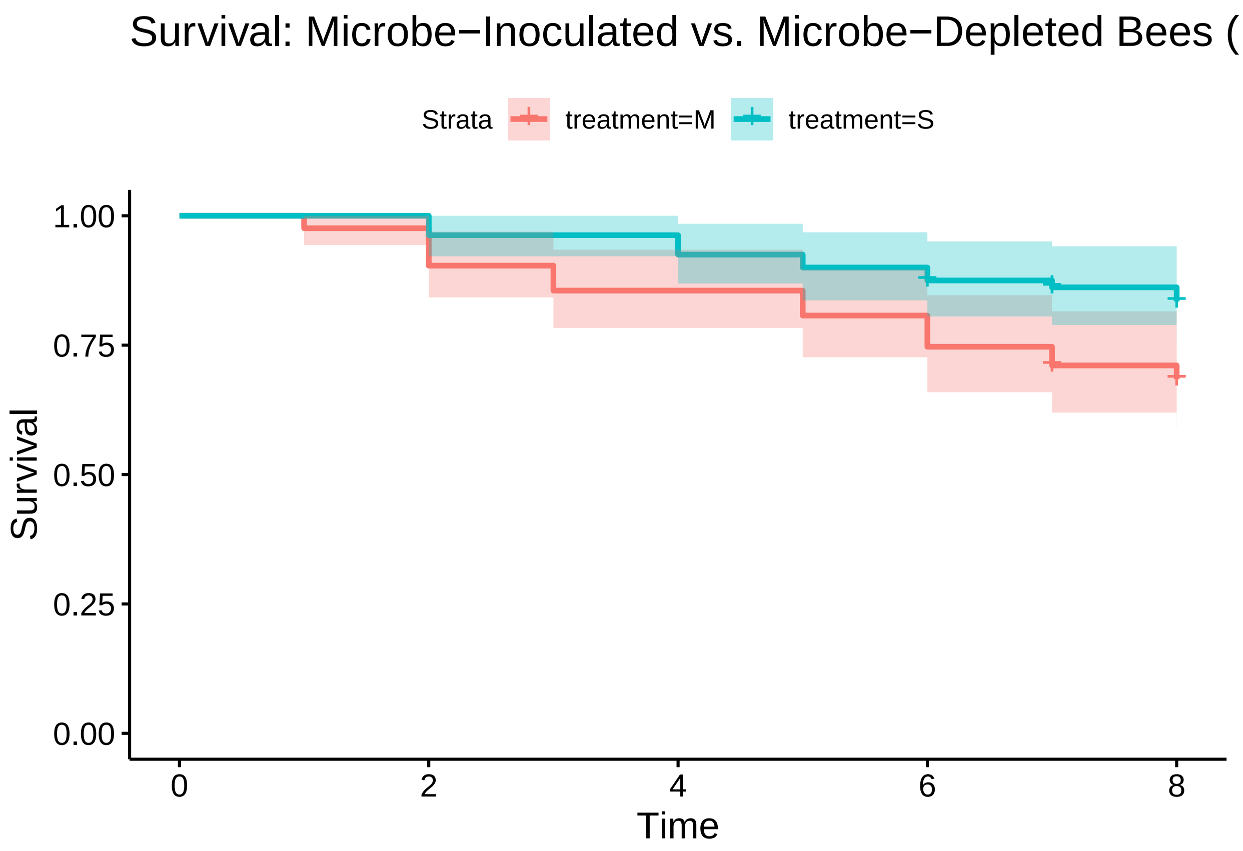

```
ggsurvplot(r1_fit, conf.int=TRUE, title=expression(paste("Survival: Microbe-Inoculated vs. Microbe-Deple
```

```
## Warning: Duplicated aesthetics after name standardisation: size
```

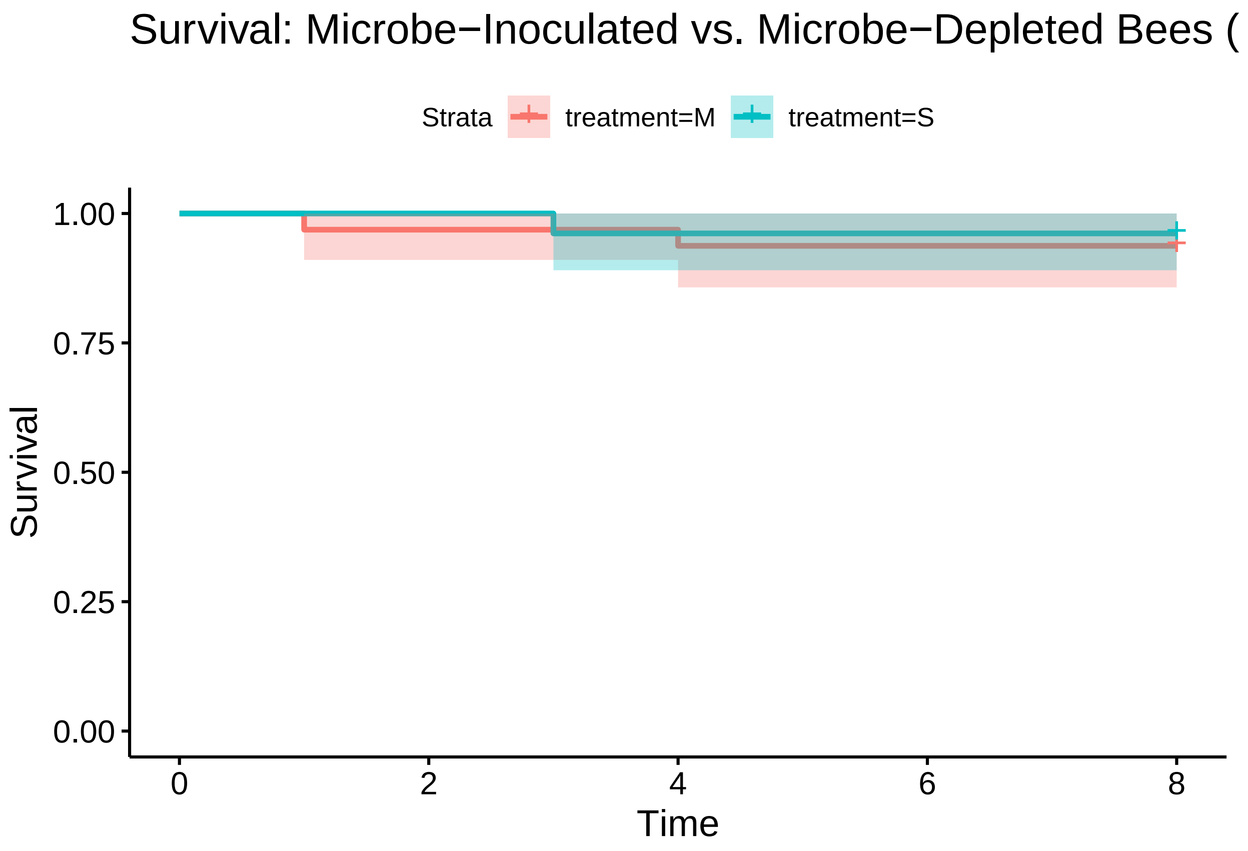

Supplement: Supplementary file 1 [file insects-11-00517-s001.zip › FMPER_bee_cognition_stats_final.pdf]
